# Forecasting the care needs of the older population in England over the next 20 years: estimates from the Population Ageing and Care Simulation (PACSim) modelling study

**DOI:** 10.1016/S2468-2667(18)30118-X

**Published:** 2018-08-31

**Authors:** Andrew Kingston, Adelina Comas-Herrera, Carol Jagger

**Affiliations:** a Institute of Health & Society and Newcastle University Institute for Ageing, Newcastle University, Newcastle, UK; b Personal Social Services Research Unit, London School of Economics and Political Science, London, UK

## Abstract

**Background:**

Existing models for forecasting future care needs are limited in the risk factors included and in the assumptions made about incoming cohorts. We estimated the numbers of people aged 65 years or older in England and the years lived in older age requiring care at different intensities between 2015 and 2035 from the Population Ageing and Care Simulation (PACSim) model.

**Methods:**

PACSim, a dynamic microsimulation model, combined three studies (Understanding Society, the English Longitudinal Study of Ageing, and the Cognitive Function and Ageing Study II) to simulate individuals' sociodemographic factors, health behaviours, 12 chronic diseases and geriatric conditions, and dependency (categorised as high [24-h care], medium [daily care], or low [less than daily] dependency; or independent). Transition probabilities for each characteristic were estimated by modelling state changes from baseline to 2-year follow-up. Years in dependency states were calculated by Sullivan's method.

**Findings:**

Between 2015 and 2035 in England, both the prevalence of and numbers of people with dependency will fall for young-old adults (65–74 years). For very old adults (≥85 years), numbers with low dependency will increase by 148·0% (range from ten simulations 140·0–152·0) and with high dependency will almost double (increase of 91·8%, range 87·3–94·1) although prevalence will change little. Older adults with medium or high dependency and dementia will be more likely to have at least two other concurrent conditions (increasing from 58·8% in 2015 to 81·2% in 2035). Men aged 65 years will see a compression of dependency with 4·2 years (range 3·9–4·2) of independence gained compared with life expectancy gains of 3·5 years (3·1–4·1). Women aged 65 years will experience an expansion of mainly low dependency, with 3·0 years (3·0–3·6) gained in life expectancy compared with 1·4 years (1·2–1·4) with low dependency and 0·7 years (0·6–0·8) with high dependency.

**Interpretation:**

In the next 20 years, the English population aged 65 years or over will see increases in the number of individuals who are independent but also in those with complex care needs. This increase is due to more individuals reaching 85 years or older who have higher levels of dependency, dementia, and comorbidity. Health and social care services must adapt to the complex care needs of an increasing older population.

**Funding:**

UK Economic and Social Research Council and the National Institute for Health Research.

## Introduction

The world's population is ageing, and many countries are finding the consequent demand on health-care services to be a challenge. Social care needs of older people (ie, aged ≥65 years) are driven by their inability to self-care and live independently, most often assessed by needing help to undertake one or more basic activities of daily living (ADLs) such as bathing, dressing, or toileting. Explicitly taking account of cognitive status and incontinence, both strong predictors of admission to long-term care, produces a more nuanced measure of dependency,^1^ and one that is closer to the WHO's concept of intrinsic capacity.^2^ Past trends in this dependency measure for the UK between 1991 and 2011 suggest increases in both low dependency (care required less than daily), from 28·7% to 32·4%, and high dependency (24-h care), from 3·9% to 5·9%.^3^ These are consistent with trends in disability-free life expectancy (as measured through ADLs) in both the UK^4^ and the USA,^5^ both of which reveal an expansion of mild disability for women.

Forecasts of care needs or dependency are often inferred from moderate or severe disability, even though considerable differences in the intensity of care are required across this range, or by using administrative data on care receipt, which measures demand rather than need. Additionally, models often fail to include risk factors other than sex, race, or education, and rarely include more than a few diseases, despite disability often being a consequence of multimorbidity^6^ and despite the numbers of older people with four or more diseases being projected to more than double between 2015 and 2035.^7^ A further limitation is that models have to make assumptions about the disability prevalence of younger cohorts ageing into the older population—generally that prevalence is similar to that of earlier cohorts—thereby ignoring differences in risk factors such as dementia, cognitive impairment, and other disabling conditions and in lifestyle and sociodemographic factors. Some of these risk factors have changed positively (dementia)^8^, ^9^ whereas others have changed negatively (obesity),^10^ and evidence suggests that the prevalence of multimorbidity in younger cohorts entering the older population will rise with each successive cohort.^7^

**Research in context**

**Evidence before this study**

Between Jan 1 and Jan 15, 2018, we searched MEDLINE and Web of Science for worldwide studies published in English from inception up to the date of the search forecasting future disability or care needs with the search terms “disability”, “life expectancy”, “longevity”, “forecast”, and “simulation”. The complete search strings and a review of the simulation models found are given in the appendix. We identified five dynamic microsimulation models, four studies forecasting disability, and one on use of social care. Only one of the studies reported years with care needs (inferred from disability) for England, and this study included only two chronic conditions (cardiovascular disease and dementia) as risk factors for disability.

**Added value of this study**

The Population Ageing and Care Simulation (PACSim) model is the first dynamic microsimulation model forecasting dependency profiles of future older populations for England based on longitudinal data from three nationally representative studies of adults aged 35 years or older and accounting for a wide range of sociodemographic and lifestyle factors and chronic conditions, as well as the real risk factor profiles of new entrants into the older population. We find that, over the next 20 years, absolute numbers of people aged 65 years or older with high dependency will increase by 36%. By 2035, most (80%) older people with medium or high dependency and dementia will also have two or more other diseases. Trends for men and women will differ markedly, with men aged 65 years seeing a compression in years lived with dependency whereas women will see an expansion of years lived with low and high dependency.

**Implications of all the available evidence**

Our projections highlight the importance of ensuring that health and social care services adapt so that they can adequately respond to the needs of an increasing older population with complex care needs; notably, this increase will probably be accompanied by a reduction in care provision by adult children as the retirement age is extended and an increase in older spouse carers who will be increasingly living with disabilities and multiple conditions.

In England, there is an ongoing debate as to the level of funding for an individual's care that should be provided by the state. Crucial to this debate is how care needs are likely to change for future cohorts of older people, in order to plan for and resource appropriate services to meet demand. We report results from a dynamic microsimulation model, the Population Ageing and Care Simulation (PACSim), to address many of the limitations of previous models, with the aim to provide the first estimates of future levels of dependency for the English population aged 65 years or older from 2015 to 2035, accounting for differences in disease and risk factor profiles of younger cohorts ageing into future older populations.

## Methods

### Model

We used PACSim to estimate the numbers with, prevalence of, and years lived with varying levels of dependency for the population aged 65 years or older in England from 2015 to 2035. Full details of the architecture of PACSim are available elsewhere;^11^ in brief, PACSim is a discrete-time dynamic microsimulation model that simulates characteristics (sociodemographic, health behaviours, chronic diseases, geriatric conditions, and dependency) of individuals. Broadly, PACSim is in two parts: creation of the base population and simulation of the ageing of individuals.

### Base population

To create the base population for PACSim, we pooled individuals aged 35 years or older from three longitudinal studies: Understanding Society wave 1 (n=27 293), the English Longitudinal Study of Ageing (ELSA) wave 5 (n=8744), and the Cognitive Function and Ageing Study II (CFAS II; n=5286), thereby ensuring a nationally representative sample of individuals who would age into the older population (≥65 years) and maximising the strengths and minimising the limitations of any one study in terms of the measured characteristics. Limitations of each study in terms of the characteristics are given elsewhere,^11^ and baseline characteristics available for all studies (by age group and study) are shown graphically in the appendix (pp 18–21). The study-specific sample weights were used to adjust for differential non-response or low response and then the pooled dataset was reweighted up to the English population in 2014 (the base of the projections), cloned so that all individuals had unit weights, and a 1% random sample taken to ease computing power and time. This formed the base population for PACSim (n=303 588).

With the exception of dementia, chronic diseases (coronary heart disease, stroke, hypertension, diabetes, arthritis, cancer, respiratory disease, and depression) were self-report of doctor diagnoses. Vision and hearing impairments were self-report of current condition and cognition status was defined by the Mini-Mental State Examination (MMSE) score,^12^ categorised as 0–9 (severe cognitive impairment), 10–20 (moderate cognitive impairment), 21–26 (mild cognitive impairment), or 27–30 (normal cognition). Dementia status was only available in CFAS II participants; for the remainder, dementia status was allocated probabilistically and outside of the simulation, conditional on age group, MMSE category, and community or care home residence. Full details of data harmonisation and imputation of missing values are given elsewhere.^11^

To measure dependency, we used the so-called interval of need developed by Isaacs and Neville.^1^ This method categorises individuals according to the frequency with which they need care: high dependency (needs 24-h care), medium dependency (needs help at regular times daily), low dependency (needs help less than daily), or independent (free from care). For ELSA and CFAS II, interval-of-need categories were constructed according to the need for help with basic and instrumental ADLs, cognitive impairment, and continence status (table 1); there were no comparable items to define dependency in the Understanding Society study and therefore the interval of need was imputed using the chained equations method^13^ on the basis of age, sex, and education. Further details of the interval-of-need classification are available elsewhere.^11^

**Table 1:** Interval-of-need dependency categorisation

	**CFAS II**	**ELSA**
High dependency	MMSE score 0–9 or needs help using the toilet, or transferring from chair or bed, or incontinent and needs help putting on shoes and socks, or needs help to feed (from proxy interview) or is often incontinent and needs help to dress (from proxy interview)	Needs help using the toilet or chairfast or bedfast or has problems with continence and needs help putting on shoes and socks
Medium dependency	Needs help every, or most days, to put on shoes and socks, or cook a hot meal, or unable to dress without help (from proxy interview)	Needs help putting on shoes and socks, or to prepare a hot meal
Low dependency	Needs help to wash all over or bathe, or cut toenails, or do heavy housework, or shopping or light housework, or considerable difficulty with household tasks (from proxy interview)	Needs help with bathing or showering, or difficulty pulling or pushing large objects, or difficulty doing work around house and garden
Independent	Not otherwise classified above and no missing items from other categories	Not otherwise classified above and no missing items from other categories

CFAS=Cognitive Function and Ageing Study. ELSA=English Longitudinal Study of Ageing. MMSE=Mini-Mental State Examination.

### Simulation

Each characteristic for an individual was updated monthly (over the period January, 2014, to December, 2042) if the probability of transition from the current value of the characteristic exceeded a randomly generated uniformly distributed variable. Transition probabilities for each stochastic characteristic (all except age, sex, education, and occupation) were calculated from fitting generalised linear models to the baseline and subsequent 2-year follow-up from the three studies pooled. All models used study-specific weights to adjust for non-response and sampling strategy. We included age and sex in all transition models and then included known risk factors for that characteristic, retaining those that were significantly associated with the transition; for dependency, we included disease and sociodemographic risk factors identified in two systematic reviews.^14^, ^15^ The transition probabilities were converted to monthly probabilities to achieve a more realistic modelling of characteristics that jointly influence each other. To account for cognitive impairment being included in the interval of need and being an explanatory factor for transitions, we modelled transitions for physical dependency (without cognitive impairment) and then combined this outcome with cognitive impairment to produce an interval of need at the end of each simulation. For low, medium, and high physical dependency; mild and moderate cognitive impairment; depression; and visual and hearing impairment, we also modelled recovery to the next less severe category. Details of the explanatory factors included in each transition model are provided in the appendix and elsewhere;^11^ dementia was excluded as an explanatory variable because it was allocated after the simulation. Death was simulated from monthly survival probabilities derived from the annual probabilities of all-cause mortality underlying the 2014-based principal population projection for England.^16^

### Model validation

PACSim well represented the time trends in the numbers of older people within broad age groups (65–74 years, 75–84 years, and ≥85 years) from the 2014 population projections (appendix). Validation against external data sources was difficult because the base population of PACSim included all the major national longitudinal studies. However, there was generally good agreement between the age-specific and sex-specific prevalence of stroke, diabetes, current smoking, overweight, and obesity from PACSim and those from the Health Survey for England 2014,^17^ apart from the prevalence of obesity, which PACSim underestimated by around 8 percentage points for men aged 35–64 years and for women of all ages.

### Model outputs

We present the prevalence of and absolute numbers with dependency for the years 2015, 2025, and 2035 by age group (65–74 years, 75–84 years, and ≥85 years), and examine the impact of dementia and multimorbidity (defined as two or more chronic conditions) on absolute numbers and prevalence estimates. Years with and without dependency at age 65 years for men and women were calculated for each year by Sullivan's method,^18^ applying the age-sex-specific prevalence of dependency to the age-sex-specific lifetable population generated from the survival probabilities. We present results from the first run of PACSim over the time period 2014–35 along with the range of values from ten repeated simulations.

Data harmonisation for the three studies was undertaken in Stata version 12.1 and PACSim was implemented in SAS version 9.4.

### Role of the funding source

This work forms part of the MODEM project. No funding body has had any influence over the design of PACSim; the collection, analysis or interpretation of data; or the writing of the report. The corresponding author had full access to all data in the study and had final responsibility for the decision to submit for publication.

## Results

Between 2015 and 2035, the absolute numbers of people aged 65 years or older in England will increase by 48·6% while the numbers living independently will increase by 61·0% overall and by more than 50% across all age groups, with the largest increase being in the very old (age ≥85 years; table 2). For the young-old (aged 65–74 years), both the numbers with and the prevalence of all levels of dependency will fall (table 2; appendix p 4). For the very old, numbers with low dependency will more than double and numbers with high dependency will almost double (table 2) owing to population increase in that age group, although dependency prevalence will change little.

**Table 2:** Projected numbers of people aged 65 years or older in England with dependency

	**Projected numbers (thousands)**			**Relative change**	
	2015	2025	2035	2015–25	2015–35
**65–74 years**					
Independent	3655 (3644 to 3669)	4493 (4491 to 4530)	5602 (5602 to 5634)	22·9% (22·8 to 23·9)	53·3% (53·3 to 54·6)
Low dependency	1144 (1144 to 1168)	806 (789 to 808)	967 (949 to 969)	−30·0% (−32·0 to −30·0)	−15·0% (−18·0 to −15·0)
Medium dependency	193 (183 to 193)	130 (124 to 133)	98 (96 to 105)	−33·0% (−33·0 to −27·0)	−49·0% (−49·0 to −43·0)
High dependency	284 (281 to 285)	248 (235 to 250)	241 (229 to 246)	−13·0% (−17·0 to −12·0)	−15·0% (−20·0 to −13·0)
**75–84 years**					
Independent	1591 (1589 to 1605)	2535 (2506 to 2537)	2778 (2768 to 2803)	59·3% (56·2 to 59·6)	74·6% (72·5 to 76·4)
Low dependency	1084 (1077 to 1100)	1213 (1213 to 1251)	1400 (1380 to 1412)	11·9% (11·9 to 15·2)	29·2% (25·7 to 30·0)
Medium dependency	189 (175 to 189)	200 (189 to 202)	171 (167 to 186)	5·7% (5·7 to 11·8)	−9·4% (−9·4 to 2·8)
High dependency	266 (265 to 272)	317 (309 to 325)	378 (371 to 385)	19·3% (15·8 to 21·0)	42·0% (36·6 to 42·7)
**≥85 years**					
Independent	295 (290 to 297)	360 (357 to 374)	539 (527 to 555)	21·9% (21·9 to 26·3)	82·6% (79·2 to 88·4)
Low dependency	621 (614 to 630)	916 (901 to 920)	1537 (1513 to 1553)	47·6% (43·1 to 48·8)	148·0% (140·0 to 152·0)
Medium dependency	169 (166 to 173)	179 (171 to 185)	293 (282 to 297)	5·9% (−1·1 to 8·7)	72·9% (65·4 to 75·9)
High dependency	233 (229 to 237)	309 (297 to 309)	446 (434 to 446)	32·9% (27·8 to 32·9)	91·8% (87·3 to 94·1)
**All ≥65 years**					
Independent	5541 (5535 to 5567)	7388 (7370 to 7419)	8918 (8913 to 8967)	33·3% (32·6 to 34·1)	61·0% (60·6 to 62·0)
Low dependency	2849 (2840 to 2882)	2934 (2929 to 2958)	3904 (3861 to 3909)	3·0% (2·1 to 4·1)	37·1% (34·9 to 37·2)
Medium dependency	552 (523 to 552)	509 (491 to 513)	562 (549 to 581)	−7·8% (−9·0 to −3·5)	1·9% (1·9 to 9·6)
High dependency	783 (778 to 790)	875 (846 to 875)	1065 (1040 to 1065)	11·8% (8·0 to 11·8)	36·0% (32·6 to 36·0)

Data in parentheses are range from ten simulations.

**Figure 1:**
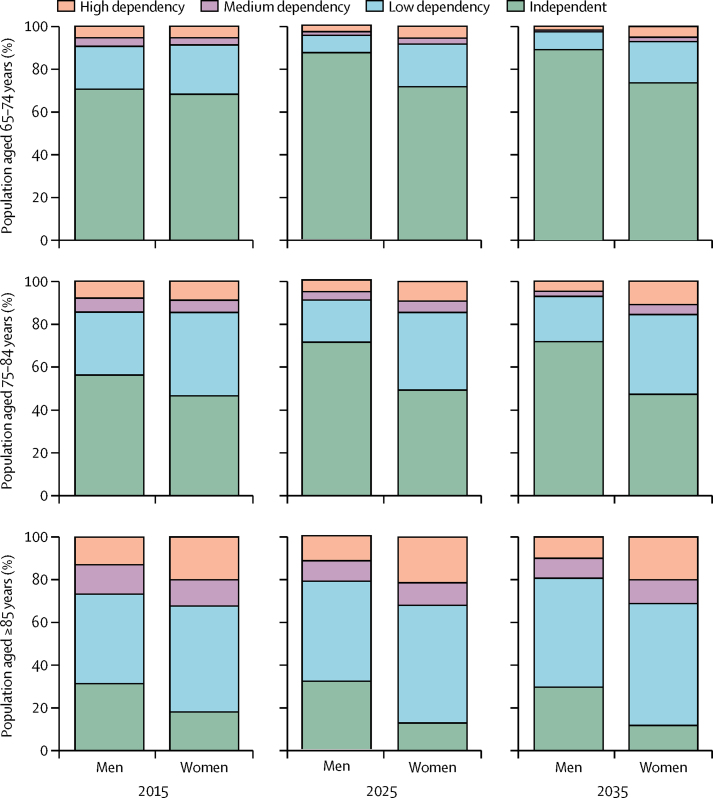
Proportion of older population who are independent or with low, medium, or high dependency by sex and age group in 2015, 2025, and 2035

The next 20 years show considerable differences by sex in the change in numbers of dependent older men and women and the prevalence of dependency (figure 1; appendix pp 5–7). In 2015, profiles of dependency in young-old men and women are similar, but by 2035, the proportion of young-old men who are independent will rise by almost 20 percentage points (from 70·5% to 89·1%), whereas the proportion of young-old women who are independent will increase by only around 6 percentage points (from 68·1% to 73·6%; figure 1). In the very old, the proportion with low dependency will rise between 2015 and 2035 for both men (from 42·1% to 51·1%) and women (from 49·7% to 57·2%), although in men this increase is mostly offset by a reduction in medium dependency (from 13·8% to 9·4%), whereas in women it is offset by a lower proportion being independent (from 17·8% to 11·5%; figure 1; appendix p 7).

**Figure 2:**
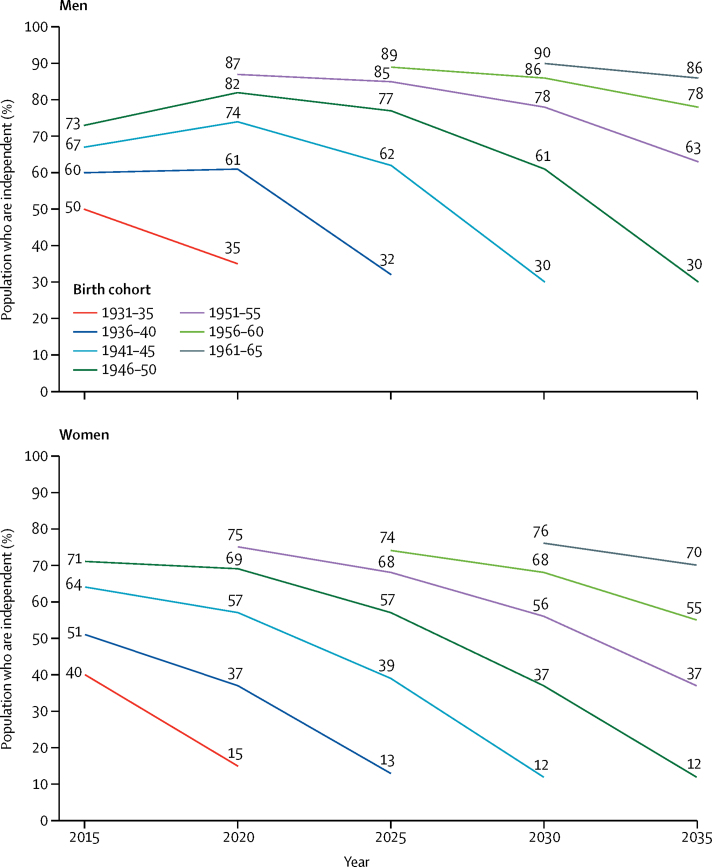
Proportion of older population who are independent up to 2035 by 5-year birth cohort and sex

The sex differences in dependency over time are seen more clearly by examining the proportion of each 5-year birth cohort who remain independent as they age (figure 2). Three phenomena are apparent. First, more recent male birth cohorts enter the older population successively more independent than prior cohorts. Second, these improvements in independence do not carry through as the male cohorts age, because the proportion of each cohort that is still independent by age 85 years or older is close to 30% regardless of birth cohort. Finally, the male cohort aged 65–69 years in 2015 (ie, those born in 1946–50) exhibits evidence of recovery of independence in the next 5 years, with the proportion who are independent at age 70–74 years in 2020 being higher than that for the same individuals at age 65–69 years in 2015; this is also true for the 70–74 year olds in 2015 (ie, those born in 1941–45) when they become 75–79 years of age in 2020. More recent male birth cohorts show no evidence of recovery. These phenomena are not evident for women, whose profile of dependency for incoming cohorts varies little and who always have a lower probability of recovery (figure 2). The proportions with low, medium, and high dependency by birth cohort are provided in the appendix (pp 12–14).

Because care needs are greatest for those with substantial (ie, moderate or high) dependency, we examined how the burden of dementia with and without other comorbidity would change over the next 20 years. Between 2015 and 2025, the composition of the older population with substantial dependency will change markedly; numbers of older adults with dementia alone will reduce by 31·1%, then remain stable up to 2035, whereas numbers of those with dementia and two or more other comorbidities will more than double by 2025 and then increase further by 2035 (table 3). Thus, by 2035, 81·2% of substantially dependent older adults with dementia will also have two or more other conditions, compared with 58·8% in 2015 (appendix p 9). Trends in multimorbidity in individuals with substantial dependency but without dementia are in the same direction but less extreme. The distributions of comorbidity in those with substantial dependency with and without dementia were similar by sex although absolute numbers were greater for women than for men in all categories (appendix pp 9–11).

**Table 3:** Projected numbers of adults in England aged 65 years or older who have substantial (medium or high) dependency with and without dementia and other comorbidities

	**Projected number (thousands)**			**Relative change**	
	2015	2025	2035	2015–25	2015–35
**Substantial dependency with dementia**					
Dementia alone	51 (51 to 60)	35 (29 to 37)	35 (32 to 38)	−31·1% (−50·5 to −31·1)	−30·5% (−45·1 to −30·5)
1 other disease	116 (108 to 116)	111 (109 to 123)	134 (130 to 142)	−4·6% (−4·6 to 8·8)	15·6% (13·5 to 28·2)
≥2 other diseases	239 (234 to 244)	512 (496 to 512)	732 (718 to 732)	114·3% (104·3 to 115·0)	206·5% (198·9 to 210·8)
**Substantial dependency without dementia**					
0–1 disease	366 (358 to 369)	144 (139 to 148)	88 (78 to 88)	−60·6% (−61·7 to −58·9)	−76·1% (−78·2 to −75·5)
2 diseases	270 (256 to 270)	191 (185 to 203)	161 (161 to 174)	−29·2% (−30·3 to −22·4)	−40·4% (−40·4 to −32·9)
≥3 diseases	293 (282 to 293)	391 (368 to 392)	477 (468 to 486)	33·4% (28·3 to 35·4)	63·0% (62·8 to 70·3)
Substantial dependency (all)*					
0–1 disease	417 (415 to 426)	179 (171 to 184)	123 (113 to 125)	−57·0% (−59·2 to −55·6)	−70·5% (−73·0 to −70·0)
2 diseases	387 (364 to 387)	302 (301 to 325)	295 (295 to 313)	−21·8% (−21·8 to −13·0)	−23·6% (−23·6 to −15·3)
≥3 diseases	532 (518 to 535)	902 (864 to 902)	1209 (1190 to 1215)	69·8% (65·6 to 69·8)	127·4% (126·6 to 132·2)

Data in parentheses are range from ten simulations.

* Dementia is included as a potential disease.

Life expectancy at age 65 years for men in England will increase between 2015 and 2035 by 3·5 years, with a gain in years independent of 4·2 years and a reduction in years with medium dependency of 0·4 years and in years of high dependency of 0·3 years (table 4). An absolute compression in the number of years lived dependent for men aged 65 years will therefore occur, with the proportion of life spent independent increasing to 2025 then decreasing slightly to 2035. However, women's life expectancy at age 65 years will increase less than for men, with almost half of this increase with low dependency and increases of 0·9 years independent and 0·7 years with high dependency (table 4). The proportion of life spent with any level of dependency changes by less than 2 percentage points for women over the whole period (table 4).

**Table 4:** Years lived from age 65 years independent and with low, medium, and high dependency, by calendar year and sex

		**Years lived from age 65**			**Change over period**	
		2015	2025	2035	2015–25	2015–35
**Men**						
Total life expectancy, years		18·7 (18·3 to 19·0)	20·7 (20·5 to 21·0)	22·2 (21·7 to 22·4)	2·0 (1·8 to 2·4)	3·5 (3·1 to 4·1)
Independent						
	Years	11·1 (10·9 to 11·3)	14·5 (14·4 to 14·6)	15·2 (15·1 to 15·2)	3·5 (3·3 to 3·5)	4·2 (3·9 to 4·2)
	Proportion of total life expectancy	59·3% (59·3 to 60·2)	70·2% (69·7 to 70·4)	68·7% (67·9 to 69·5)	10·9% (9·7 to 10·9)	9·4% (8·0 to 9·7)
Low dependency						
	Years	5·0 (4·8 to 5·1)	4·2 (4·1 to 4·3)	5·1 (4·8 to 5·3)	−0·9 (−0·9 to −0·6)	0·1 (−0·1 to 0·4)
	Proportion of total life expectancy	26·9% (26·2 to 26·9)	20·0% (20·0 to 20·7)	23·0% (22·4 to 23·7)	−6·8% (−6·8 to −5·8)	−3·9% (−4·3 to −2·9)
Medium dependency						
	Years	1·2 (1·1 to 1·2)	0·9 (0·8 to 0·9)	0·8 (0·7 to 0·8)	−0·3 (−0·4 to −0·3)	−0·4 (−0·4 to −0·3)
	Proportion of total life expectancy	6·4% (6·1 to 6·4)	4·1% (3·8 to 4·1)	3·5% (3·4 to 3·7)	−2·2% (−2·4 to −2·0)	−2·9% (−2·9 to −2·4)
High dependency						
	Years	1·4 (1·4 to 1·4)	1·2 (1·1 to 1·2)	1·1 (1·0 to 1·1)	−0·2 (−0·2 to −0·2)	−0·3 (−0·4 to −0·3)
	Proportion of total life expectancy	7·5% (7·4 to 7·6)	5·6% (5·5 to 5·8)	4·8% (4·7 to 4·9)	−1·8% (−2·0 to −1·7)	−2·6% (−2·8 to −2·6)
**Women**						
Total life expectancy, years		21·1 (20·8 to 21·1)	22·7 (22·5 to 23·3)	24·1 (23·9 to 24·4)	1·7 (1·5 to 2·2)	3·0 (3·0 to 3·6)
Independent						
	Years	10·7 (10·5 to 10·7)	11·4 (11·3 to 11·5)	11·6 (11·6 to 11·8)	0·7 (0·7 to 0·9)	0·9 (0·9 to 1·2)
	Proportion of total life expectancy	50·6% (50·1 to 50·7)	49·9% (49·4 to 50·3)	48·0% (48·0 to 48·6)	−0·6% (−0·8 to 0·2)	−2·6% (−2·6 to −1·6)
Low dependency						
	Years	7·2 (7·1 to 7·3)	7·7 (7·6 to 8·0)	8·5 (8·4 to 8·6)	0·5 (0·3 to 0·7)	1·4 (1·2 to 1·4)
	Proportion of total life expectancy	34·0% (34·0 to 34·7)	33·9% (33·6 to 34·3)	35·4% (35·0 to 35·4)	−0·1% (−0·9 to 0·0)	1·4% (0·4 to 1·4)
Medium dependency						
	Years	1·3 (1·2 to 1·3)	1·3 (1·2 to 1·3)	1·3 (1·3 to 1·4)	0·0 (0·0 to 0·1)	0·0 (0·0 to 0·2)
	Proportion of total life expectancy	6·0% (5·5 to 6·0)	5·5% (5·5 to 5·7)	5·4% (5·2 to 5·6)	−0·5% (−0·5 to 0·2)	−0·6% (−0·6 to −0·1)
High dependency						
	Years	2·0 (1·9 to 2·0)	2·4 (2·3 to 2·5)	2·7 (2·6 to 2·8)	0·4 (0·3 to 0·5)	0·7 (0·6 to 0·8)
	Proportion of total life expectancy	9·5% (9·3 to 9·6)	10·7% (10·2 to 10·7)	11·2% (10·8 to 11·3)	1·2% (0·8 to 1·2)	1·7% (1·3 to 2·0)

Total life expectancy is given as years of life remaining. Data in parentheses are range from ten simulations.

## Discussion

We report the first forecasts of levels of dependency in adults aged 65 years or older in England that include the sociodemographic and disease profiles for cohorts becoming 65 years of age over the period 2015–35. Our overall findings are that the proportion of independent older people will increase between 2015 and 2035 although absolute numbers with low or high dependency will still rise by around a third. However, trends for men and women will be very different. Over the period studied, men (aged 65–74 years) will be successively more independent when they enter the older population, with the proportion independent increasing by almost 20 percentage points between 2015 and 2035. Nevertheless, the improved dependency is not sustained, and once they reach 85 years of age or older they are indistinguishable from earlier cohorts. Young-old women (aged 65–74 years) will also see an increase in the proportion independent, but only by around 6 percentage points, and with almost no change in profiles of dependency between 2025 and 2035. Men aged 65 years will experience a real compression of dependency, with years of independence gained exceeding the gain in life expectancy. Women, on the other hand, will have most of the gain in life expectancy in years with low dependency, and with a small increase in years with high dependency, identical to trends over the previous two decades.^3^

Dementia incidence and prevalence have fallen over the past decades in the UK and the USA,^8^, ^9^ but projections suggest that dementia prevalence will rise owing to longer survival and the increasing numbers of the very old.^7^, ^19^ Due to the growth in the numbers of very old adults and the greater prevalence of multimorbidity in this age group, most older adults with medium or high dependency and dementia in 2035 will also have other comorbidities. This group is likely to have more complex care needs that are unlikely to be met adequately without improved coordination between different specialities and without improved recognition of the way in which a diagnosis of dementia affects the management of other long-term conditions.^20^

The need to inform policy and resource allocation for care of future older populations is evident from other dynamic microsimulation models for England, Japan, New Zealand, the USA, and Canada (see appendix [pp 23–25] for a review of simulation models). PACSim addresses the limitations of previous microsimulation models: care needs inferred only from ADL disability,^21^, ^22^, ^23^ use of social care,^24^ or a combination;^25^ assumptions about risk factor and dependency profiles for new entrants to the older population rather than inclusion of real individuals as they age into the older population;^21^, ^22^, ^24^, ^25^ omission of key sociodemographic and lifestyle factors, and chronic conditions that impact disability and dependency;^22^, ^23^, ^24^, ^25^ and the inability to observe the joint effect of diseases or multimorbidity. These differences between models are illustrated by comparison of our findings with those of the previous model for England, IMPACT-BAM,^22^ based on one of the studies (ELSA) included in PACSim. IMPACT-BAM reports a greater increase in years with disability for men than for women between 2015 and 2025. By contrast, PACSim forecasts a reduction in years of dependence (all levels) for men over the same period, although the disability and dependency measures are not directly comparable, the latter including cognitive impairment and instrumental ADLs, which provide a wider spectrum of functional limitation. Other strengths of PACSim are the use of three large, nationally representative surveys as its base population, with the baseline disease prevalence broadly comparable with the Health Survey for England 2014;^17^ incorporation of level of education, which has increased—and will continue to increase for some time—in the older population; and performing the simulation on a monthly schedule, thus providing a more realistic evolution of individual characteristics that are co-dependent and the ability to transition rapidly through the severity of dependency. Limitations to our study concern the assumptions inherent in PACSim: that the transition models remain constant over time, although the transition probabilities will change over time if the risk factor profile of cohorts change and these can be further modified through exploring different scenarios; that the ADLs, cognitive status, and incontinence defining dependency levels will do so in the same way over the next 20 years, which might not be the case because technological advances might replace carer intervention; and omission of other risk factors such as alcohol use.

Several diseases that increase the risk of dependency share obesity as an underlying risk factor. Worldwide, there have been increasing trends in obesity and overweight,^26^ and halting the rise in obesity and diabetes is one of the global targets for non-communicable diseases for WHO.^27^ However, other risk factors for dependency have improved over past decades with reductions in stroke incidence^28^ and in dementia prevalence^9^ reported in the USA and reductions in dementia incidence reported in the UK.^8^ Nevertheless, even with reductions in incidence or prevalence of chronic conditions, the ageing of the population means a substantial increase in the number of older people with long-term conditions and with multiple concurrent conditions because multimorbidity is the norm for very old people, who are the fastest growing demographic;^29^ however, population ageing might explain only up to half of past rises in multimorbidity prevalence.^30^ In the future, although young-old cohorts are more likely to enter old age independent, the proportion of the population who are multimorbid is forecast to rise with each successive cohort,^7^ and these result in a lower likelihood of recovery of independence, particularly in men, and therefore a greater likelihood of higher dependency with further ageing.

Our forecasts highlight the importance of ensuring that health and social care services adapt so that they can adequately respond to the needs of an increasing older population with complex care needs.^2^ The rise in care needs we have forecast contrasts with other trends that suggest that relying on families and other unpaid carers more than done so currently does not appear to be a sustainable solution. The supply of unpaid care to older people by their adult children in England is unlikely to keep pace with demand,^31^ whereas care provision by spouses is growing and is projected to continue to increase in importance.^32^ Older spouse carers are increasingly likely to be living with disabilities themselves, resulting in mutual care relationships^33^ that are not yet well recognised by existing care policy and practices. Extending the retirement age of the UK population is likely to further reduce the informal and unpaid carer pool, who have traditionally provided for older family members, and so shift this responsibility to the state.^34^ These forces will unite to add further stress to social care budgets that help people to maintain independence within the community or fund long-term care needs.
