# Supplementary material for: Forecasting the care needs of the older population in England over the next 20 years: estimates from the Population Ageing and Care Simulation (PACSim) modelling study
Source: Lancet Public Health. 2018 Aug 31;3(9):e447–55. doi: 10.1016/S2468-2667(18)30118-X (PMC6123499; doi:10.1016/S2468-2667(18)30118-X)
Supplement: Supplementary appendix [file mmc1.pdf]

THE LANCET
Public Health

Supplementary appendix

This appendix formed part of the original submission and has been peer reviewed. We post it as supplied by the authors.

Supplement to: Kingston A, Comas-Herrera A, Jagger C. Forecasting the care needs of the older population in England over the next 20 years: estimates from the Population Ageing and Care Simulation (PACSim) modelling study. *Lancet Public Health* 2018; published online Aug 30. http://dx.doi.org/10.1016/S2468-2667(18)30118-X.

Supplementary appendix

This appendix formed part of the original submission and has been peer reviewed.

Supplement to: Kingston A, Comas-Herrera A, Jagger C, for the MODEM project. Forecasting the care needs of the older population in England over the next 20 years: estimates from the Population Ageing and Care Simulation (PACSim) modelling study. *Lancet Public Health* 2018; published online Aug 30. http://dx.doi.org/10.1016/S2468-2667(18)30118-X

Contents

Results 4

Table S.1: Prevalence of dependency of older adults in England in 2015, 2025 and 2035, by age.... 4

Table S.2: Projected numbers of older men in England with dependency (in thousands) in 2015, 2025 and 2035, by age 5

Table S.3: Projected numbers of older women in England with dependency (in thousands) in 2015, 2025 and 2035, by age 6

Table S.4: Prevalence of dependency of older adults in England in 2015, 2025 and 2035, by age and gender. 7

Table S.5: Projected proportion (%) of older adults aged ≥65 years in England with substantial (moderate or high) dependency with and without dementia who have other comorbidity, 2015, 2025 and 2035 8

Table S.6: Projected proportion (%) of older men and women aged ≥65 years in England with substantial (moderate or high) dependency with and without dementia who have other comorbidity, 2015, 2025 and 2035 9

Table S.7: Projected numbers (thousands) of older men aged ≥65 years in England who have substantial (moderate or high) dependency with and without dementia and other comorbidity, 2015, 2025 and 2035 10

Table S.8: Projected numbers (thousands) of older women aged ≥65 years in England who have substantial (moderate or high) dependency with and without dementia and other comorbidity, 2015, 2025 and 2035 11

Figure S.1: Proportion of older individuals with low dependency in England in 2015, 2020, 2025, 2030, 2035 by five year birth cohort and sex 12

Figure S.2: Proportion of older individuals with medium dependency in England in 2015, 2020, 2025, 2030, 2035 by five year birth cohort and sex 13

Figure S.3: Proportion of older individuals with high dependency in England in 2015, 2020, 2025, 2030, 2035 by five year birth cohort and sex 14

Methods 15

Base population 15

Transition probabilities 15

Model validation 15

Table S.9: Modelled transition relationships between variables 16

Table S.10: Life expectancies at age 65 from PACSim (with range from repeated simulations) and from ONS 2014 projections, 2015, 2025, 2035 by sex 17

Figure S.4: Comparison of baseline proportion with low education and proportion of current smokers by study and age group 18

Figure S.5: Comparison of baseline proportion with hypertension, coronary heart disease (CHD), stroke, and diabetes by study and age group 19

Figure S.6: Comparison of baseline proportion with arthritis, respiratory disease, cancer, and depression by study and age group 20

Figure S.7: Comparison of baseline proportion with hearing and visual impairment by study and age group 21
Figure S.8: Comparison of number of individuals in England by age group and year from PACSim with ONS 2014-based population projections 22
Research in context 23
Table S.11: Results of literature review on dynamic microsimulation models 24
References 25

Results

Table S.1: Prevalence of dependency of older adults in England in 2015, 2025 and 2035, by age.

	**2015**	**2025**	**2035**
65-74 years			
Independent	69.3 (69.0-69.5)	79.2 (79.1-79.7)	81.1 (81.1-81.4)
Low dependency	21.7 (21.7-22.1)	14.2 (13.9-14.2)	14.0 (13.7-14.0)
Medium dependency	3.7 (3.5-3.7)	2.3 (2.2-2.3)	1.4 (1.4-1.5)
High dependency	5.4 (5.3-5.4)	4.4 (4.1-4.4)	3.5 (3.3-3.5)
75-84 years			
Independent	50.8 (50.7-51.2)	59.4 (58.8-59.5)	58.8 (58.5-59.2)
Low dependency	34.6 (34.4-35.1)	28.4 (28.4-29.3)	29.6 (29.2-29.7)
Medium dependency	6.0 (5.6-6.0)	4.7 (4.4-4.7)	3.6 (3.5-3.9)
High dependency	8.5 (8.4-8.7)	7.4 (7.2-7.6)	8.0 (7.9-8.1)
≥85 years			
Independent	22.4 (22.2-22.6)	20.4 (20.3-21.2)	19.1 (18.9-19.9)
Low dependency	47.1 (46.7-47.5)	51.9 (51.3-52.3)	54.6 (54.1-55.3)
Medium dependency	12.9 (12.6-13.1)	10.2 (9.7-10.4)	10.4 (10.0-10.6)
High dependency	17.7 (17.4-18.0)	17.5 (16.9-17.5)	15.9 (15.4-15.9)
≥65 years			
Independent	57.0 (56.9-57.2)	63.1 (63.0-63.3)	61.7 (61.7-62.0)
Low dependency	29.3 (29.2-29.6)	25.1 (25.0-25.3)	27.0 (26.7-27.0)
Medium dependency	5.7 (5.4-5.7)	4.3 (4.2-4.4)	3.9 (3.8-4.0)
High dependency	8.1 (8.0-8.1)	7.5 (7.2-7.5)	7.4 (7.2-7.4)

Data in parentheses are minimum and maximum from ten simulations

Table S.2: Projected numbers of older men in England with dependency (in thousands) in 2015, 2025 and 2035, by age

				relative change (%)	
**MEN**	**2015**	**2025**	**2035**	**2015-2025**	**2015-2035**
65-74 years					
independent	1794 (1794-1808)	2399 (2384-2412)	2983 (2969-2993)	33.7 (31.9-34.3)	66.3 (64.8-66.3)
low dependency	512 (506-521)	224 (220-231)	280 (266-287)	-56.0 (-58.0--55.0)	-45.0 (-48.0--45.0)
medium dependency	101 (94-101)	47 (42-47)	27 (24-30)	-53.0 (-56.0--51.0)	-73.0 (-74.0--68.0)
high dependency	137 (135-138)	84 (84-90)	60 (58-65)	-39.0 (-39.0--33.0)	-56.0 (-57.0--52.0)
75-79 years					
independent	784 (784-811)	1405 (1371-1405)	1595 (1573-1595)	79.2 (70.3-79.2)	103.0 (94.2-103.0)
low dependency	411 (404-411)	389 (389-412)	472 (470-490)	-5.4 (-5.4-2.0)	14.9 (14.6-21.0)
medium dependency	91 (84-91)	79 (72-79)	54 (53-61)	-12.0 (-17.0--8.1)	-40.0 (-40.0--30.0)
high dependency	111 (108-114)	106 (105-110)	105 (105-114)	-4.2 (-6.3--0.2)	-5.6 (-6.0-4.3)
≥85 years					
independent	142 (140-148)	223 (222-237)	352 (342-364)	57.1 (54.8-66.2)	147.0 (135.0-156.0)
low dependency	192 (183-194)	330 (322-336)	609 (596-630)	71.6 (66.9-82.6)	217.0 (208.0-243.0)
medium dependency	63 (62-66)	68 (62-71)	112 (107-115)	7.6 (-5.5-13.4)	77.0 (66.9-83.6)
high dependency	60 (58-62)	82 (76-83)	119 (111-119)	38.0 (26.0-42.5)	101.0 (79.0-101.0)
All ≥65 years					
independent	2720 (2720-2765)	4027 (4001-4036)	4930 (4897-4931)	48.0 (45.0-48.0)	81.2 (77.4-81.2)
low dependency	1116 (1098-1119)	943 (943-970)	1361 (1353-1387)	-15.0 (-15.0--12.0)	22.0 (21.5-24.9)
medium dependency	254 (241-254)	194 (179-194)	193 (190-201)	-24.0 (-28.0--21.0)	-24.0 (-24.0--18.0)
high dependency	307 (305-311)	272 (268-281)	284 (281-293)	-11.0 (-13.0--9.2)	-7.6 (-9.6--5.2)

Data in parentheses are minimum and maximum from ten simulations

Table S.3: Projected numbers of older women in England with dependency (in thousands) in 2015, 2025 and 2035, by age

				relative change (%)	
**WOMEN**	**2015**	**2025**	**2035**	**2015-2025**	**2015-2035**
65-74 years					
independent	1861 (1847-1861)	2095 (2095-2120)	2619 (2619-2645)	12.6 (12.6-14.4)	40.8 (40.8-43.2)
low dependency	632 (632-647)	582 (566-582)	687 (676-694)	-7.9 (-11.0--7.9)	8.7 (6.2-8.9)
medium dependency	93 (88-93)	82 (82-88)	71 (71-79)	-11.0 (-11.0--0.9)	-23.0 (-23.0--11.0)
high dependency	147 (146-149)	165 (151-165)	181 (168-183)	11.7 (2.7-11.7)	22.9 (15.1-22.9)
75-79 years					
independent	807 (787-807)	1130 (1122-1138)	1182 (1182-1214)	40.0 (40.0-43.9)	46.6 (46.6-53.6)
low dependency	673 (673-694)	824 (814-840)	928 (908-937)	22.5 (19.0-23.1)	38.0 (32.2-38.0)
medium dependency	99 (89-99)	121 (117-126)	117 (114-125)	22.2 (22.2-38.4)	18.8 (18.8-38.2)
high dependency	155 (155-161)	211 (203-215)	273 (259-276)	36.2 (28.1-37.2)	76.1 (65.0-76.6)
≥85 years					
independent	153 (147-154)	136 (131-139)	187 (185-198)	-11.0 (-13.0--6.6)	22.4 (22.4-33.7)
low dependency	428 (428-436)	586 (576-592)	928 (906-931)	36.8 (32.4-36.8)	117.0 (108.0-117.0)
medium dependency	106 (101-108)	112 (109-115)	181 (169-182)	4.9 (1.6-13.1)	70.5 (62.2-74.0)
high dependency	173 (167-176)	227 (219-227)	327 (323-329)	31.2 (25.1-33.7)	88.7 (85.9-95.4)
All ≥65 years					
independent	2820 (2794-2820)	3361 (3361-3389)	3989 (3989-4044)	19.2 (19.2-21.3)	41.4 (41.4-44.6)
low dependency	1733 (1733-1768)	1991 (1965-1999)	2543 (2489-2545)	14.9 (11.9-14.9)	46.8 (41.8-46.8)
medium dependency	298 (278-298)	315 (312-324)	370 (359-380)	5.6 (5.6-16.4)	24.2 (24.2-33.9)
high dependency	476 (469-483)	603 (577-603)	781 (753-781)	26.8 (21.8-26.8)	64.2 (58.4-64.2)

Data in parentheses are minimum and maximum from ten simulations

Table S.4: Prevalence of dependency of older adults in England in 2015, 2025 and 2035, by age and gender.

	2015	2025	2035
Men 65-74 years			
Independent	70.5 (70.5-71.0)	87.1 (86.7-87.4)	89.1 (88.9-89.4)
Low dependency	20.1 (19.9-20.4)	8.1 (8.0-8.4)	8.4 (8.0-8.6)
Medium dependency	4.0 (3.7-4.0)	1.7 (1.5-1.7)	0.8 (0.7-0.9)
High dependency	5.4 (5.3-5.4)	3.0 (3.0-3.3)	1.8 (1.7-1.9)
Women 65-74 years			
Independent	68.1 (67.6-68.2)	71.7 (71.7-72.4)	73.6 (73.6-74.0)
Low dependency	23.1 (23.1-23.7)	19.9 (19.3-19.9)	19.3 (18.9-19.4)
Medium dependency	3.4 (3.2-3.4)	2.8 (2.8-3.0)	2.0 (2.0-2.2)
High dependency	5.4 (5.3-5.5)	5.6 (5.1-5.6)	5.1 (4.7-5.1)
Men 75-84 years			
Independent	56.1 (56.1-57.5)	71.0 (69.8-71.0)	71.7 (70.6-71.7)
Low dependency	29.4 (28.7-29.4)	19.7 (19.7-20.9)	21.2 (21.0-21.9)
Medium dependency	6.5 (5.9-6.5)	4.0 (3.6-4.0)	2.4 (2.4-2.8)
High dependency	7.9 (7.6-8.1)	5.4 (5.3-5.5)	4.7 (4.7-5.1)
Women 75-84 years			
Independent	46.5 (45.6-46.5)	49.4 (49.2-49.9)	47.3 (47.3-48.5)
Low dependency	38.8 (38.8-40.1)	36.1 (35.6-36.6)	37.1 (36.2-37.1)
Medium dependency	5.7 (5.2-5.7)	5.3 (5.1-5.5)	4.7 (4.5-5.0)
High dependency	8.9 (8.9-9.3)	9.2 (8.9-9.4)	10.9 (10.4-11.0)
Men ≥85 years			
Independent	31.1 (30.8-32.4)	31.8 (31.6-33.4)	29.5 (28.6-30.7)
Low dependency	42.1 (40.1-42.1)	46.9 (45.9-47.8)	51.1 (50.4-52.7)
Medium dependency	13.8 (13.5-14.2)	9.6 (8.8-9.9)	9.4 (9.0-9.7)
High dependency	13.0 (12.8-13.8)	11.7 (10.9-11.8)	10.0 (9.3-10.0)
Women ≥85 years			
Independent	17.8 (17.2-17.8)	12.8 (12.4-13.2)	11.5 (11.5-12.2)
Low dependency	49.8 (49.7-50.8)	55.2 (54.9-55.7)	57.2 (56.3-57.7)
Medium dependency	12.4 (11.8-12.5)	10.5 (10.3-10.9)	11.2 (10.5-11.3)
High dependency	20.1 (19.4-20.5)	21.4 (20.9-21.6)	20.1 (20.0-20.5)
Men ≥65 years			
Independent	61.9 (61.9-62.6)	74.1 (73.7-74.1)	72.8 (72.4-72.9)
Low dependency	25.4 (24.9-25.4)	17.3 (17.3-17.8)	20.1 (20.0-20.5)
Medium dependency	5.8 (5.5-5.8)	3.6 (3.3-3.6)	2.8 (2.8-3.0)
High dependency	7.0 (6.9-7.1)	5.0 (4.9-5.2)	4.2 (4.2-4.3)
Women ≥65 years			
Independent	52.9 (52.5-52.9)	53.6 (53.6-54.1)	51.9 (51.9-52.7)
Low dependency	32.5 (32.5-33.2)	31.8 (31.4-31.8)	33.1 (32.4-33.1)
Medium dependency	5.6 (5.2-5.6)	5.0 (5.0-5.2)	4.8 (4.7-4.9)
High dependency	8.9 (8.8-9.1)	9.6 (9.2-9.6)	10.2 (9.8-10.2)

Data in parentheses are minimum and maximum from ten simulations

Table S.5: Projected proportion (%) of older adults aged ≥65 years in England with substantial (moderate or high) dependency with and without dementia who have other comorbidity, 2015, 2025 and 2035

	2015	2025	2035
Substantial dependency with dementia			
Dementia alone	12.5 (12.5 - 14.8)	5.3 (4.5 - 5.7)	3.9 (3.6 - 4.2)
With 1 other disease	28.6 (26.6 - 28.6)	16.9 (16.9 - 18.6)	14.9 (14.6 - 15.7)
With 2+ other diseases	58.8 (57.7 - 58.8)	77.8 (76.3 - 77.8)	81.2 (80.2 - 81.6)
Substantial dependency without dementia			
0-1 disease	39.4 (39.3 - 40.4)	19.9 (19.6 - 20.9)	12.1 (10.7 - 12.1)
2 diseases	29.1 (28.1 - 29.1)	26.3 (25.8 - 28.5)	22.2 (22.2 - 23.8)
3+ diseases	31.5 (31.3 - 32.1)	53.8 (51.9 - 54.1)	65.8 (64.6 - 66.4)
Substantial dependency ALL			
0-1 disease	31.2 (31.2 - 32.4)	13.0 (12.5 - 13.5)	7.5 (6.9 - 7.6)
2 diseases	29.0 (27.7 - 29.0)	21.8 (21.8 - 23.7)	18.2 (18.2 - 19.2)
3+ diseases	39.8 (39.5 - 40.5)	65.2 (63.7 - 65.2)	74.3 (73.3 - 74.7)

Data in parentheses are minimum and maximum from ten simulations

Table S.6: Projected proportion (%) of older men and women aged ≥65 years in England with substantial (moderate or high) dependency with and without dementia who have other comorbidity, 2015, 2025 and 2035

	2015	2025	2035
Substantial dependency with dementia, men			
Dementia alone	13.5 (13.5 - 18.2)	6.6 (5.2 - 6.6)	3.8 (3.2 - 4.5)
With 1 other disease	27.5 (25.3 - 27.5)	15.1 (15.1 - 20.3)	14.8 (13.3 - 15.9)
With 2+ other diseases	59.0 (56.1 - 59.0)	78.3 (74.3 - 78.3)	81.4 (80.2 - 82.6)
Substantial dependency with dementia, women			
Dementia alone	12.1 (12.1 - 13.4)	4.8 (4.2 - 5.3)	3.9 (3.5 - 4.1)
With 1 other disease	29.1 (27.1 - 29.1)	17.6 (16.5 - 18.4)	15.0 (14.2 - 16.0)
With 2+ other diseases	58.8 (57.9 - 59.5)	77.6 (76.4 - 78.8)	81.1 (80.0 - 81.9)
Substantial dependency without dementia, men			
0-1 disease	40.8 (40.3 - 41.6)	18.6 (18.5 - 20.0)	7.6 (7.3 - 8.3)
2 diseases	28.9 (28.3 - 30.1)	23.9 (22.3 - 25.9)	17.8 (16.2 - 19.1)
3+ diseases	30.2 (29.5 - 30.8)	57.5 (55.6 - 58.9)	74.6 (72.6 - 75.5)
Substantial dependency without dementia, women			
0-1 disease	38.1 (38.1 - 39.5)	20.6 (19.6 - 21.6)	14.1 (12.1 - 14.2)
2 diseases	29.3 (27.5 - 29.3)	27.8 (27.5 - 30.0)	24.2 (24.2 - 26.1)
3+ diseases	32.7 (32.5 - 33.5)	51.6 (49.8 - 52.0)	61.6 (59.8 - 62.6)
Substantial dependency ALL, men			
0-1 disease	34.9 (34.9 - 36.3)	13.6 (12.8 - 13.9)	5.7 (5.4 - 6.3)
2 diseases	28.6 (27.6 - 29.2)	20.2 (20.2 - 23.4)	16.2 (15.5 - 17.1)
3+ diseases	36.5 (35.3 - 36.7)	66.2 (63.8 - 66.2)	78.1 (76.7 - 78.5)
Substantial dependency ALL, women			
0-1 disease	28.5 (28.5 - 29.7)	12.7 (11.9 - 13.3)	8.3 (7.4 - 8.3)
2 diseases	29.2 (27.7 - 29.2)	22.7 (22.3 - 23.9)	18.9 (18.9 - 20.4)
3+ diseases	42.3 (42.1 - 43.2)	64.7 (63.2 - 64.9)	72.7 (71.3 - 73.6)

Data in parentheses are minimum and maximum from ten simulations

Table S.7: Projected numbers (thousands) of older men aged ≥65 years in England who have substantial (moderate or high) dependency with and without dementia and other comorbidity, 2015, 2025 and 2035

				Relative change (%)	
**MEN**	**2015**	**2025**	**2035**	**2015-2025**	**2015-2035**
Substantial dependency with dementia					
Dementia alone	16 (16 - 22)	13 (10 - 13)	9 (8 - 11)	-20.7 (-53.3 - -20.7)	-42.7 (-62.4 - -42.7)
With 1 other disease	33 (31 - 33)	30 (30 - 40)	36 (33 - 38)	-11.4 (-11.4 - 28.3)	8.1 (2.2 - 24.3)
With 2+ diseases	72 (68 - 72)	154 (142 - 154)	199 (194 - 207)	114.1 (100.3 - 124.4)	177.5 (173.1 - 202.2)
Substantial dependency without dementia					
0-1 disease	180 (173 - 182)	50 (48 - 53)	18 (17 - 20)	-72.0 (-73.2 - -70.0)	-90.1 (-90.5 - -88.5)
2 diseases	127 (122 - 130)	65 (59 - 68)	41 (38 - 45)	-49.2 (-52.9 - -44.2)	-67.5 (-69.3 - -64.4)
3+ diseases	133 (126 - 133)	155 (144 - 156)	173 (169 - 181)	16.8 (10.0 - 20.0)	30.0 (30.0 - 39.1)
Substantial dependency ALL					
0-1 disease	196 (194 - 202)	63 (58 - 64)	27 (26 - 30)	-67.8 (-70.5 - 0.0)	-86.2 (-86.7 - -84.6)
2 diseases	161 (154 - 162)	94 (94 - 107)	77 (74 - 82)	-41.3 (-41.3 - 0.0)	-51.8 (-51.8 - -47.8)
3+ diseases	205 (196 - 205)	309 (292 - 309)	372 (370 - 384)	50.9 (45.7 - 0.0)	81.7 (81.7 - 95.6)

Data in parentheses are minimum and maximum from ten simulations

Table S.8: Projected numbers (thousands) of older women aged ≥65 years in England who have substantial (moderate or high) dependency with and without dementia and other comorbidity, 2015, 2025 and 2035

				Relative change (%)	
**WOMEN**	**2015**	**2025**	**2035**	**2015-2025**	**2015-2035**
Substantial dependency with dementia					
Dementia alone	34 (34 - 39)	22 (19 - 24)	26 (23 - 27)	-36.0 (-48.9 - -30.9)	-24.7 (-37.4 - -23.8)
With 1 other disease	83 (77 - 83)	81 (74 - 84)	98 (93 - 105)	-1.9 (-8.0 - 5.1)	18.6 (15.7 - 32.3)
With 2+ diseases	167 (163 - 173)	358 (345 - 362)	533 (514 - 535)	114.4 (105.4 - 118.0)	219.0 (204.3 - 224.4)
Substantial dependency without dementia					
0-1 disease	186 (184 - 188)	94 (90 - 97)	70 (60 - 70)	-49.5 (-51.4 - -48.2)	-62.5 (-67.7 - -62.5)
2 diseases	143 (131 - 143)	127 (124 - 136)	120 (120 - 129)	-11.4 (-11.4 - 0.2)	-16.3 (-16.3 - -2.8)
3+ diseases	160 (154 - 161)	235 (223 - 238)	305 (293 - 311)	47.2 (39.3 - 49.4)	90.4 (81.8 - 99.9)
Substantial dependency ALL					
0-1 disease	221 (221 - 226)	116 (109 - 120)	96 (85 - 96)	-47.4 (-51.0 - -51.0)	-56.6 (-61.8 - -56.6)
2 diseases	226 (210 - 226)	208 (202 - 218)	218 (218 - 231)	-7.9 (-7.9 - -7.9)	-3.5 (-3.5 - 9.2)
3+ diseases	327 (321 - 332)	594 (569 - 594)	837 (808 - 843)	81.6 (73.0 - 73.0)	156.1 (145.4 - 157.5)

Data in parentheses are minimum and maximum from ten simulation

Figure S.1: Proportion of older individuals with low dependency in England in 2015, 2020, 2025, 2030, 2035 by five year birth cohort and sex

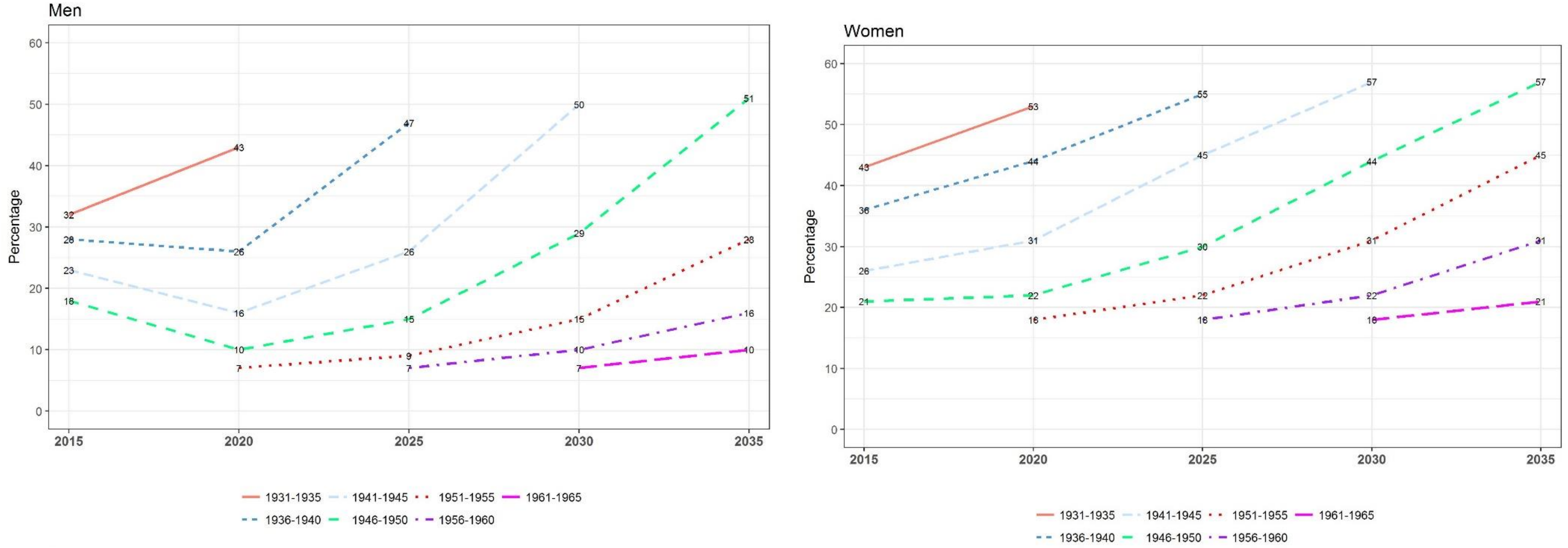

			Men						Women		
Cohort	**2015**	**2020**	**2025**	**2030**	**2035**		**2015**	**2020**	**2025**	**2030**	**2035**
1931-1935	32.1 (31.9-32.8)	43.0 (42.2-43.2)					42.9 (42.9-45.4)	52.9 (52.9-54.5)			
1936-1940	27.6 (26.0-27.6)	25.9 (25.3-26.4)	46.9 (45.9-47.8)				35.7 (34.8-36.1)	44.2 (42.7-44.2)	55.2 (54.9-55.7)		
1941-1945	22.7 (22.7-23.6)	16.2 (15.9-17.1)	26.4 (26.4-28.4)	49.8 (49.8-51.4)			26.3 (26.3-27.1)	31.3 (30.7-31.4)	44.6 (43.4-44.8)	56.9 (56.5-57.6)	
1946-1950	18.3 (17.5-18.3)	10.3 (10.3-11.1)	15.3 (15.2-16.2)	28.6 (27.9-29.0)	51.1 (50.4-52.7)		20.7 (20.5-21.2)	21.7 (21.3-22.6)	30.1 (29.9-31.0)	44.1 (43.2-44.6)	57.2 (56.3-57.7)
1951-1955		7.3 (7.0-7.4)	9.2 (9.2-9.8)	15.5 (15.1-16.0)	28.2 (27.8-28.8)			17.8 (17.2-18.4)	22.2 (21.1-22.4)	31.2 (30.3-31.2)	44.8 (44.0-44.8)
1956-1960			7.2 (6.8-7.6)	9.7 (9.3-10.1)	16.1 (15.7-16.7)				17.9 (17.4-18.3)	21.6 (21.5-22.1)	30.9 (29.6-30.9)
1961-1965				6.9 (6.4-7.0)	10.2 (9.4-10.2)					17.5 (16.8-17.5)	21.3 (20.9-21.7)

Data in parentheses are minimum and maximum from ten simulation

Figure S.2: Proportion of older individuals with medium dependency in England in 2015, 2020, 2025, 2030, 2035 by five year birth cohort and sex

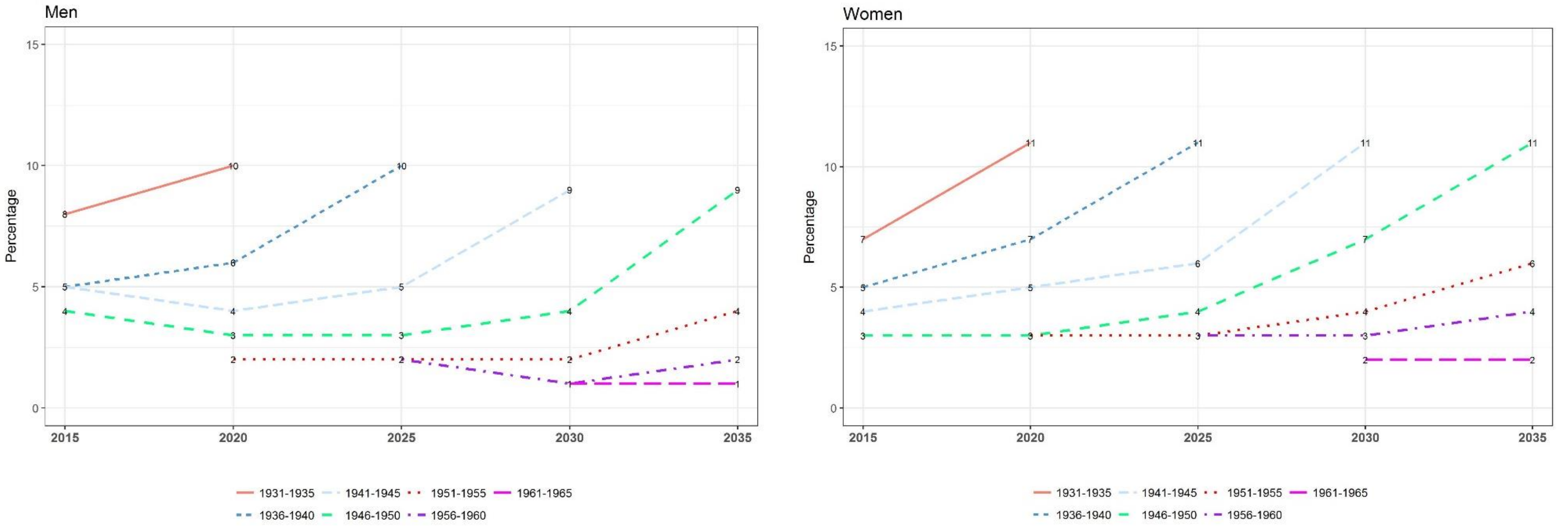

			Men						Women		
Cohort	**2015**	**2020**	**2025**	**2030**	**2035**		**2015**	**2020**	**2025**	**2030**	**2035**
1931-1935	8.2 (7.0-8.2)	10.1 (10.1-10.9)					6.9 (6.3-6.9)	11.1 (10.6-11.2)			
1936-1940	5.3 (5.0-5.5)	5.8 (5.3-5.9)	9.6 (8.8-9.9)				4.7 (4.3-4.7)	6.9 (6.7-7.2)	10.5 (10.3-10.9)		
1941-1945	4.6 (4.0-4.6)	4.2 (3.6-4.5)	5.3 (4.5-5.3)	9.4 (8.7-9.5)			4.0 (3.5-4.0)	4.7 (4.5-5.0)	6.4 (6.3-6.7)	10.7 (10.6-11.0)	
1946-1950	3.5 (3.3-3.5)	2.9 (2.6-3.1)	3.1 (2.8-3.3)	4.4 (4.1-4.5)	9.4 (9.0-9.7)		2.9 (2.8-3.1)	3.3 (3.3-3.7)	4.5 (4.2-4.7)	6.5 (6.2-6.6)	11.2 (10.5-11.3)
1951-1955		2.0 (1.9-2.2)	1.9 (1.7-1.9)	2.3 (2.2-2.4)	3.5 (3.3-3.9)			2.7 (2.6-2.8)	2.9 (2.9-3.2)	4.0 (3.5-4.1)	5.6 (5.2-6.0)
1956-1960			1.5 (1.2-1.5)	1.5 (1.3-1.5)	1.6 (1.6-2.0)				2.7 (2.7-3.0)	3.2 (2.8-3.2)	3.9 (3.9-4.2)
1961-1965				0.9 (0.6-0.9)	0.9 (0.8-1.1)					1.8 (1.8-2.2)	2.2 (2.2-2.6)

Data in parentheses are minimum and maximum from ten simulation

Figure S.3: Proportion of older individuals with high dependency in England in 2015, 2020, 2025, 2030, 2035 by five year birth cohort and sex

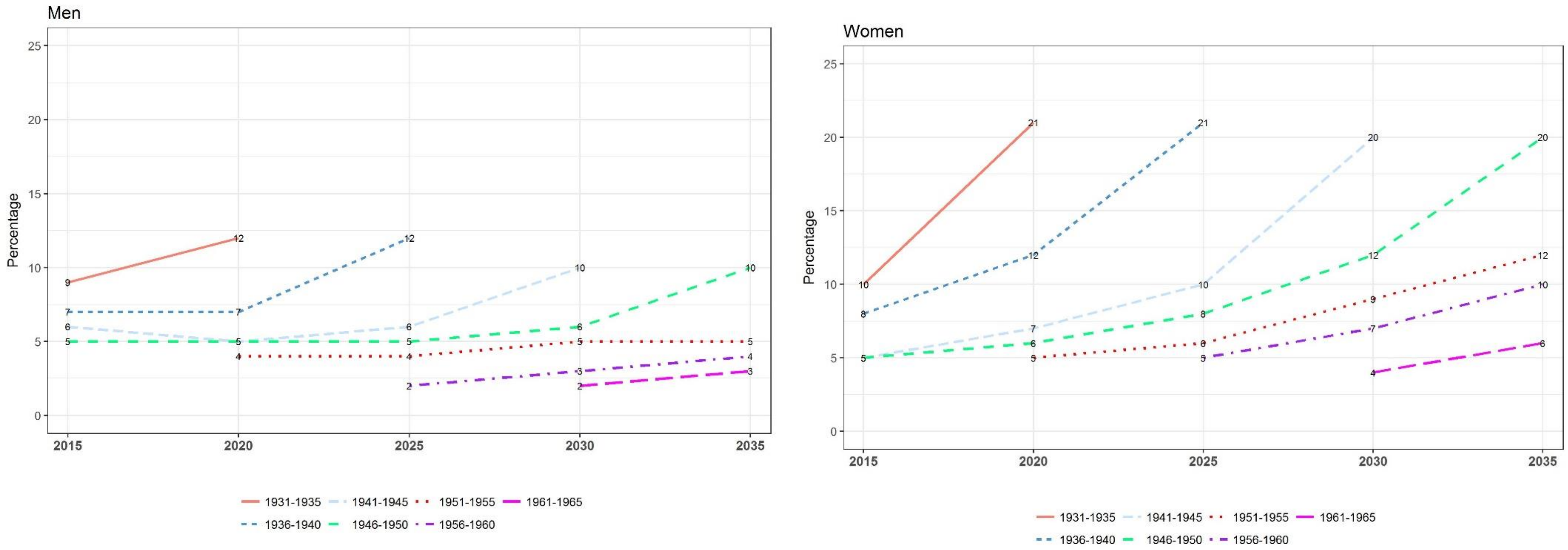

			Men					Women		
Cohort	**2015**	**2020**	**2025**	**2030**	**2035**		**2015**	**2020**	**2025**	**2030**	**2035**
1931-1935	9.4 (9.4-10.2)	12.2 (12.1-12.9)					9.8 (9.4-10.0)	21.4 (20.6-21.5)			
1936-1940	6.9 (6.4-6.9)	7.2 (6.7-7.4)	11.7 (10.9-11.8)				8.3 (8.3-8.8)	11.7 (11.5-12.3)	21.4 (20.9-21.6)		
1941-1945	5.8 (5.7-6.0)	5.5 (5.2-5.7)	6.1 (5.8-6.5)	10.4 (9.6-10.5)			5.4 (5.2-5.4)	7.3 (6.5-7.3)	10.4 (9.6-10.6)	20.3 (19.2-20.5)	
1946-1950	5.1 (4.8-5.1)	4.6 (4.3-4.8)	4.9 (4.8-5.3)	6.0 (5.9-6.2)	10.0 (9.3-10.0)		5.4 (5.3-5.6)	6.3 (6.0-6.3)	8.4 (8.2-8.7)	12.0 (11.4-12.4)	20.1 (20.0-20.5)
1951-1955		3.6 (3.4-3.7)	3.7 (3.7-4.0)	4.6 (4.4-4.8)	5.4 (5.4-6.4)			4.6 (4.2-4.6)	6.5 (5.7-6.5)	9.1 (8.2-9.1)	12.4 (11.6-12.5)
1956-1960			2.5 (2.4-2.7)	3.1 (3.0-3.4)	4.2 (4.0-4.4)				4.9 (4.6-5.2)	6.9 (6.5-7.1)	9.8 (9.2-9.8)
1961-1965				1.8 (1.5-1.8)	2.5 (2.3-2.6)					4.2 (4.0-4.3)	6.4 (6.1-6.7)

Data in parentheses are minimum and maximum from ten simulation

Methods

Base population

To enable estimates for the population aged ≥65 years to be simulated 2040, the base population for PACSim required individuals aged ≥35 years. Three longitudinal studies were pooled to construct the base population and one follow-up wave (generally around two years later) for calculation of the transition probabilities: Understanding Society (US), the English Longitudinal Study of Ageing (ELSA), and the Cognitive Function and Ageing Study II (CFAS II). US is a longitudinal study of the social and economic circumstances, attitudes, behaviours and health of people aged 16 and over in the UK; we used wave 1 (2009-10) and wave 2 (2010-11). ELSA provided data on the health, social, wellbeing and economic circumstances of the English population aged 50 and older; we used wave 5 (2010-11) for the baseline and wave 6. CFAS II aims to investigate dementia and cognitive decline in a representative sample of more than 18,000 people aged 65 years and over; only two waves have been completed, wave 1 (2008-11) and wave 2 (2011-13). Comparisons of the major characteristics available in all studies at baseline (education, smoking, all chronic conditions and sensory impairment) by age group and study are shown in Figures S.4 – S.7.

Transition probabilities

For all three datasets there was an interval of two years between waves for estimation of transition probabilities for each characteristic (apart from education and social class which were fixed). For the remaining stochastic characteristics (diseases, health behaviours, physical dependency) transition probabilities were estimated from fitting generalised linear models to the pooled dataset. Where missing values were imputed for a whole study (e.g. BMI in CFAS, cognitive impairment in Understanding Society) we excluded the study from estimation of the transition probabilities.

Most diseases were considered chronic and therefore only incidence (transition to disease) was estimated; the exception was depression where the probability of recovery was also estimated. Similarly, recovery for vision and hearing impairment were modelled in addition to transition from mild cognitive impairment (MMSE 21-26) to normal cognition (MMSE 27-30). For physical activity, there was insufficient data to model transitions, therefore the transition probabilities were approximated by fitting models to the baseline data only. Transition probabilities for BMI used ELSA waves 4 and 6 as BMI was not collected in wave 5. For ordered categorical characteristics (BMI, physical dependency, cognitive function) ordinal logistic models could not be fitted as the proportional odds assumption was violated, and therefore a generalised logit model was fitted.

The modelled transition relationships between variables are shown in Table S.9 with √ denoting a significant association. If variables (apart from age and sex) were not significant in the model, the coefficient was replaced by zero (and shown in Table S.9 by X).

Model validation

Validation against external data sources is difficult as the base population for PACSim included the three major national (or nationally representative) longitudinal studies. The number of individuals by five-year age group (65-69, 70-74, 75-79, 80-84, 85-89, 90+) and year (2014-2041) from PACSim were compared to the ONS 2014 population projections; the largest percentage difference was in the oldest age groups (90+) which reached over 2.5% between 2022 and 2025; for ages 65-84 the percentage difference in numbers of individuals between PACSim and ONS was under 1% and mostly below 0.5% throughout (Figure S.8). We also compared the estimates of forecasted life expectancy at age 65 with those from ONS (Table S.10); differences were small.

Table S.9: Modelled transition relationships between variables

	Predictors																			
	**Age**	**Sex**	**Education**	**Marital status**	**Occupation**	**Smoking**	**BMI**	**Physical activity**	**Coronary Heart Disease**	**Stroke**	**Hypertension**	**Diabetes**	**Arthritis**	**Depression**	**Respiratory**	**Cancer**	**Vision**	**Hearing**	**Cognition**	**Dependency**
**Marital status**	√	√																		
**Smoking**	√	√	X	X																
**BMI**	√	√	√																	
**Physical activity**	√	√	√				√													
**Coronary Heart Disease**	√	√				X	√	√			X	X								
**Stroke**	√	√				√	√				√									
**Hypertension**	√	√	√			X	√	X												
**Diabetes**	√	√	√			X	√	√			√									
**Arthritis**	√	√					√													
**Depression**	√	√		√				√												
**Respiratory disease**	√	√	X		X	√	√													
**Cancer**	√	√				X	X													
**Vision**	√	√	√			√														
**Hearing**	√	√	√			√														
**Cognition**	√	√	√			√	√	√		√	√	√		√			√	√		
**Dependency**	√	√	√						√	√	√	√	√	√	√	√	√	X	√	
**Retirement**	√	√	X		X				√		√		√	√	X					
**Institution**	√	√	√	√															√	√

√ = significant predictor,   X =   entered into transition model but not significant

Table S.10: Life expectancies at age 65 from PACSim (with range from repeated simulations) and from ONS 2014 projections, 2015, 2025, 2035 by sex

	Life expectancy at age 65		
	**2015**	**2025**	**2035**
**Men**			
PACSim	18.7 (18.3 - 19.0)	20.7 (20.5 - 21.0)	22.2 (21.7 - 22.4)
ONS	18.9	20.8	22.1
**Women**			
PACSim	21.1 (20.8 - 21.1)	22.7 (22.5 - 23.3)	24.1 (23.9 - 24.4)
ONS	21.2	22.9	24.1

Figure S.4: Comparison of baseline proportion with low education and proportion of current smokers by study and age group

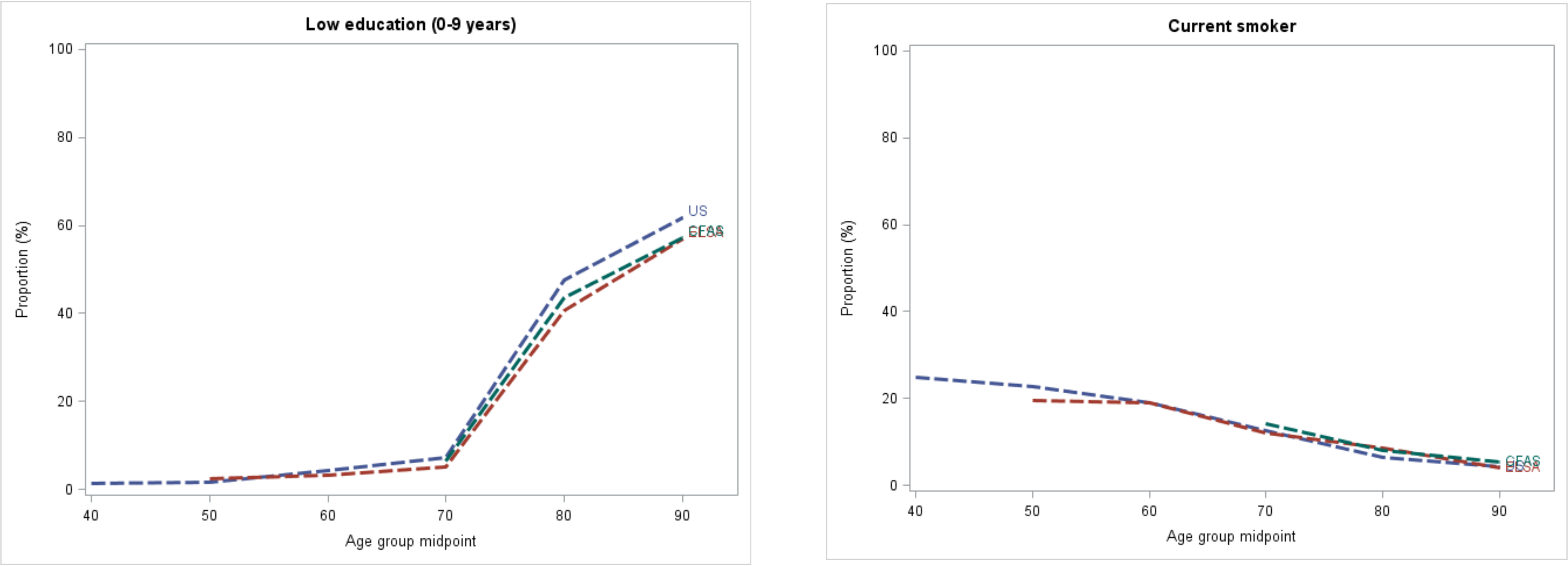

Figure S.5: Comparison of baseline proportion with hypertension, coronary heart disease (CHD), stroke, and diabetes by study and age group

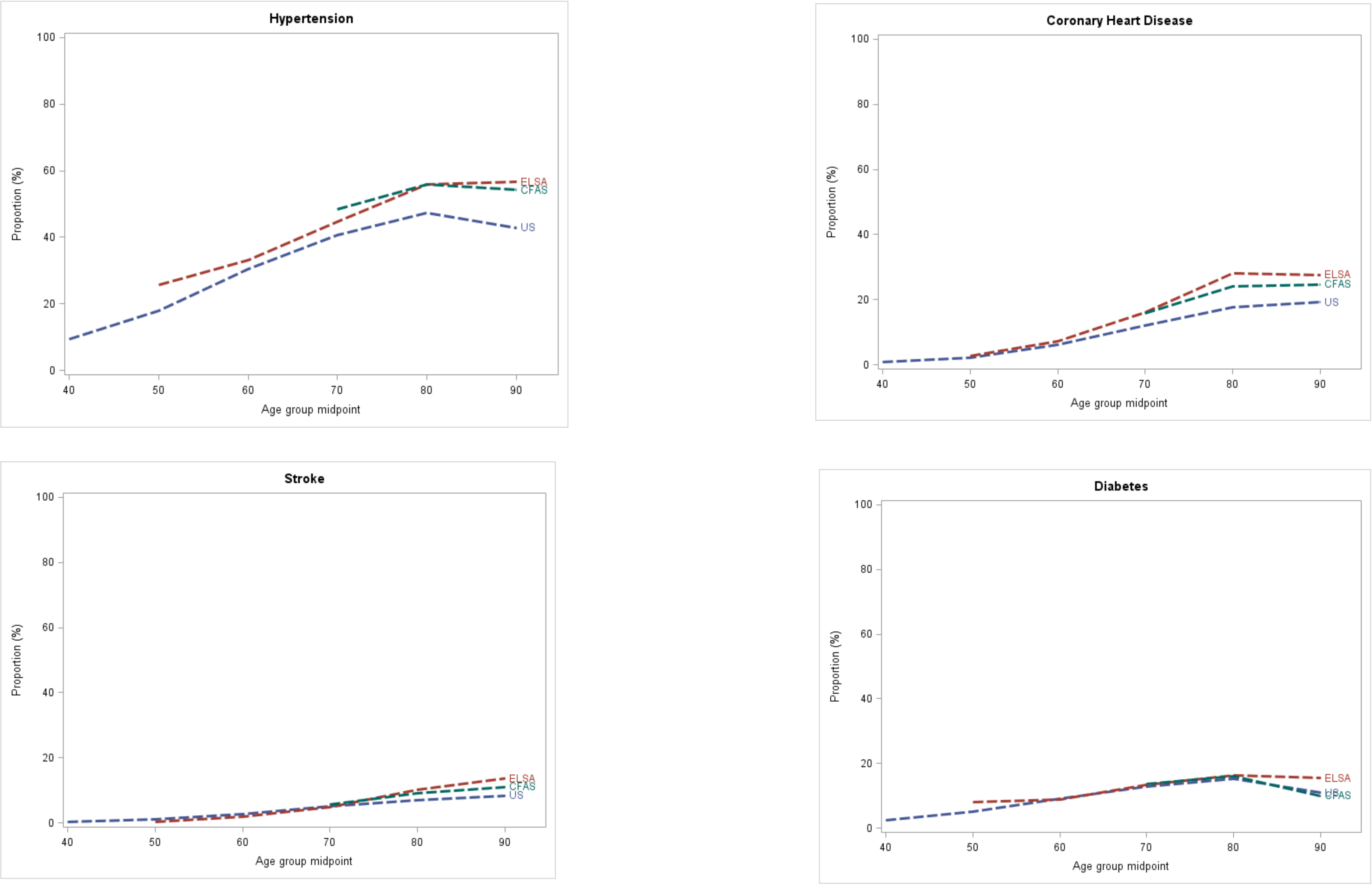

Figure S.6: Comparison of baseline proportion with arthritis, respiratory disease, cancer, and depression by study and age group

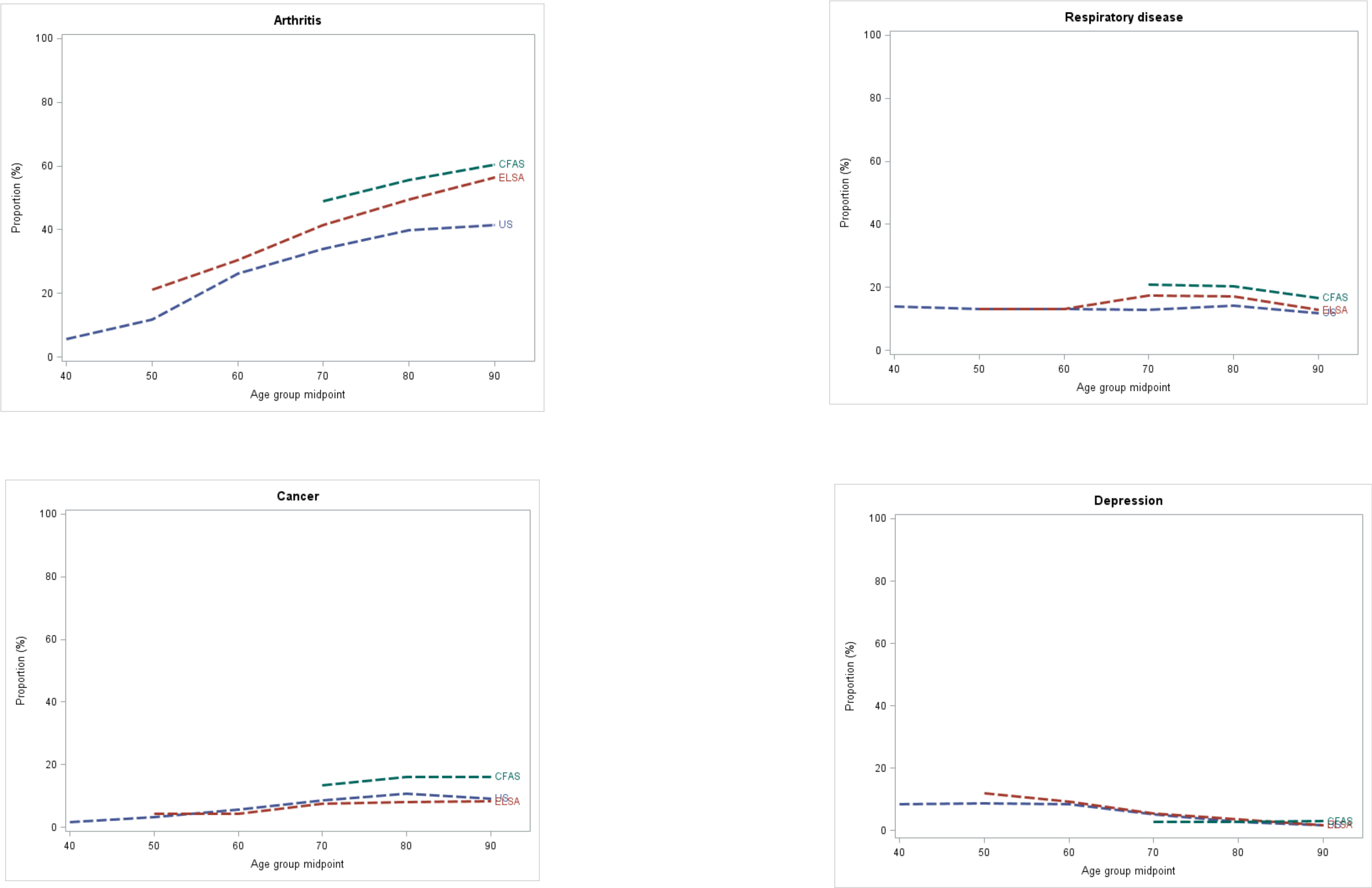

Figure S.7: Comparison of baseline proportion with hearing and visual impairment by study and age group

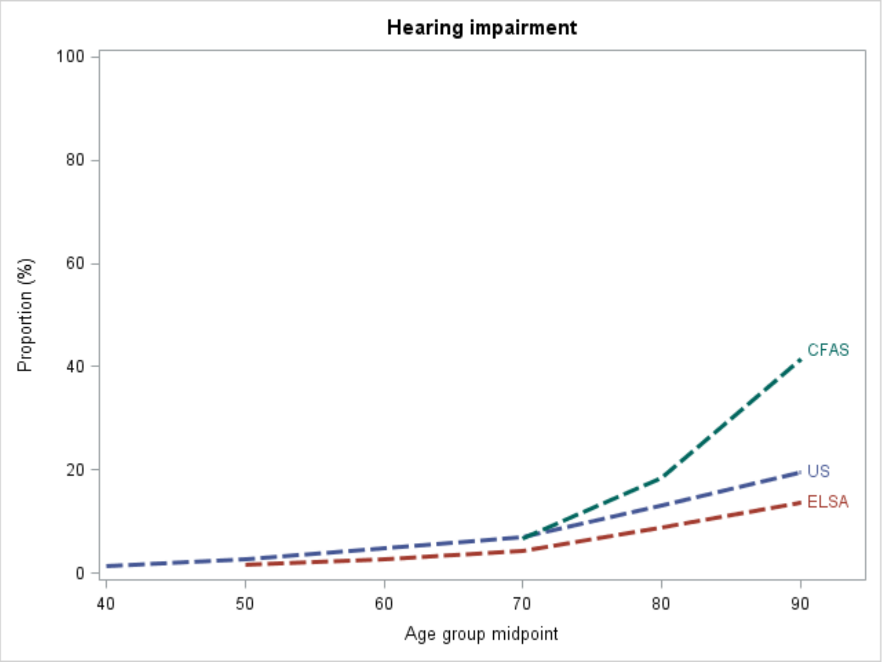

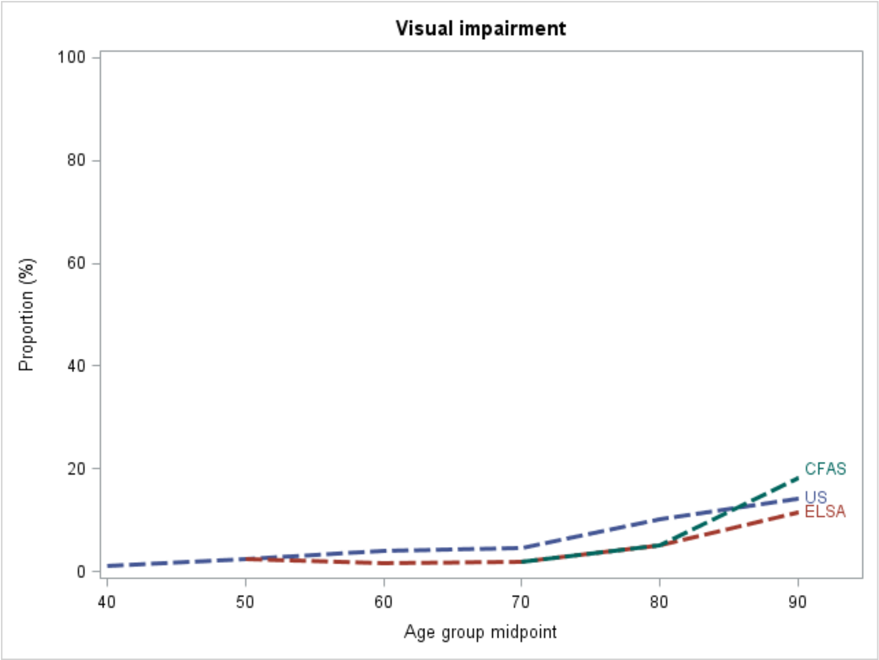

Figure S.8: Comparison of number of individuals in England by age group and year from PACSim with ONS 2014-based population projections

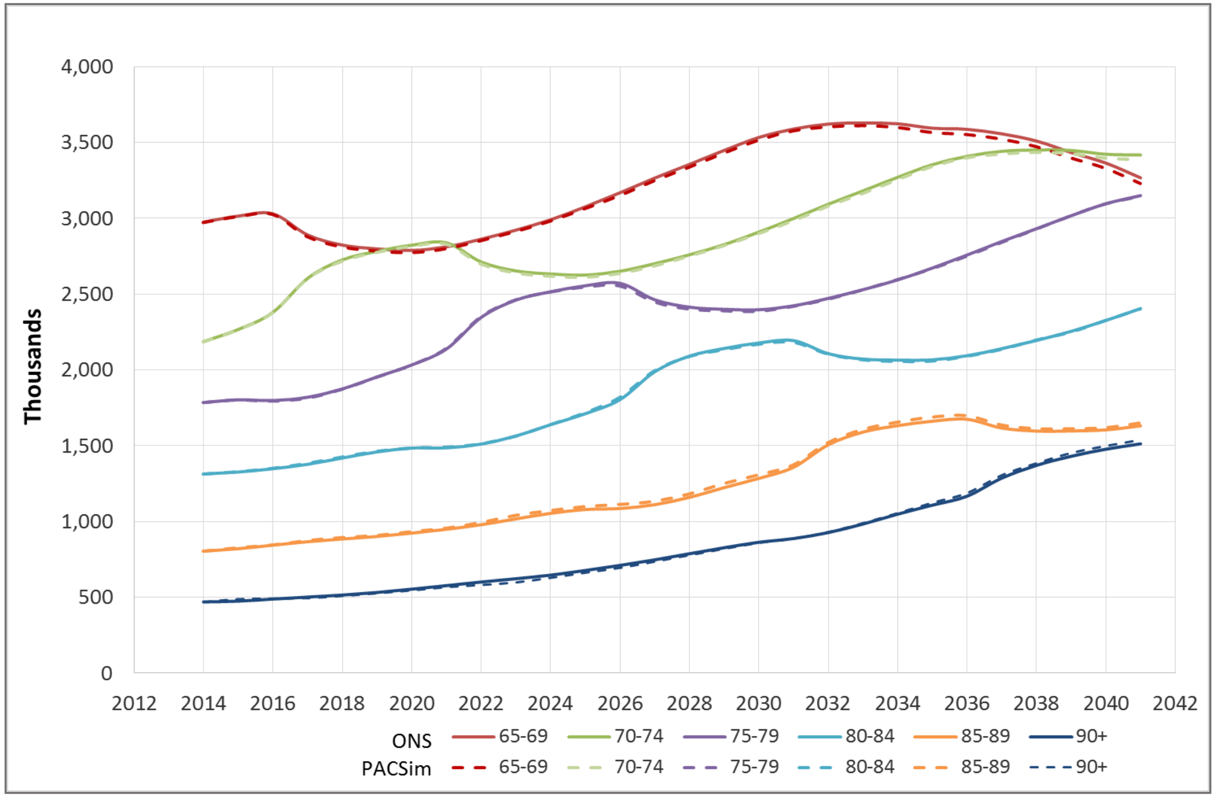

Research in context

We reviewed existing evidence in January 2018 for simulation models forecasting dependency or care needs worldwide. We searched both Medline and Web of Science with the following search terms:

("Disabled Persons"[Mesh] OR "Life Expectancy"[Mesh] OR Disab*[ti]

OR Longevit*[ti] OR Life expectan*[ti])

AND

("Computer Simulation"[Mesh] OR "Forecasting"[Mesh] OR "Population Forecast"[Mesh] OR

Simulat*[ti] OR Model*[ti] OR forecast*[ti] OR project*)

A review of abstracts identified relevant articles which were then extracted; references of relevant articles were hand searched for further articles. We focused on dynamic microsimulation models, as PACSim, and identified five such models in Canada, England, Japan, New Zealand and the United States (detailed in Table S.11).

Table S.11: Results of literature review on dynamic microsimulation models

Author	Title	Population	Age	Time horizon	Outcomes	Risk factors included
Legare et al[1]	Using microsimulation to reassess aging trends in Canada	Canada	20+	2001-2051	Disability (none, mild, moderate, severe)	education, marital status, age at immigration
Guzman-Castillo et al[2]	Forecasted trends in disability and life expectancy in England and Wales up to 2025: a modelling study	England	65+	2015-2025	ADL disability (1+)	cardiovascular disease, cognitive impairment
Chen et al[3]	Forecasting trends in disability in a super-aging society: Adapting the Future Elderly Model to Japan	Japan	50+	2014-2040	ADL disability (0,1,2,3+), IADL disability (0,1,2,3+), social functioning (0,1,2,3+), help received (yes/no)	smoking, BMI, 19 diseases
Lay-Yee et al[4]	Changing the balance of social care for older people: simulating scenarios under demographic ageing in New Zealand	New Zealand	65+	2001-2021	use of social care (informal, formal, residential)	ethnicity, deprivation level, partnership status, disability (assistance in activities of daily living)
Goldman et al[5]	Consequences of health trends and medical innovation for the future elderly	US	65+	2000-2030	disability (1+ADLs, 3+ADLs, nursing home)	education, race, ethnicity, smoking, BMI, 8 health conditions

References

1. Legare J, Decarie Y, Belanger A. Using microsimulation to reassess aging trends in Canada. *Canadian journal on aging = La revue canadienne du vieillissement* 2014; **33**(2): 208-19.

2. Guzman-Castillo M, Ahmadi-Abhari S, Bandosz P, et al. Forecasted trends in disability and life expectancy in England and Wales up to 2025: a modelling study. *The Lancet Public Health* 2017; **2**(7): e307-e13.

3. Chen BK, Jalal H, Hashimoto H, et al. Forecasting trends in disability in a super-aging society: Adapting the Future Elderly Model to Japan. *Journal of the Economics of Ageing* 2016; **8**: 42-51.

4. Lay-Yee R, Pearson J, Davis P, von Randow M, Kerse N, Brown L. Changing the balance of social care for older people: simulating scenarios under demographic ageing in New Zealand. *Health & Social Care in the Community* 2017; **25**(3): 962-74.

5. Goldman DP, Shang B, Bhattacharya J, et al. Consequences of health trends and medical innovation for the future elderly. *Health affairs (Project Hope)* 2005; **24 Suppl 2**: W5r-17.
